# Co-ingestion of Black Tea Reduces the Indispensable Amino Acid Digestibility of Hens’ Egg in Indian Adults

**DOI:** 10.1093/jn/nxz091

**Published:** 2019-05-25

**Authors:** Sindhu Kashyap, Nirupama Shivakumar, Aneesia Varkey, Thomas Preston, Sarita Devi, Anura V Kurpad

**Affiliations:** 1 Division of Nutrition, St. John's Research Institute; 2 Department of Physiology, St. John's Medical College, St. John's National Academy of Health Sciences, Bangalore, India; 3 Scottish Universities Environmental Research Centre, East Kilbride, Scotland, United Kingdom

**Keywords:** black tea, tea polyphenol, intrinsically labeled egg, true indispensable amino acid digestibility, dual isotope tracer technique

## Abstract

**Background:**

Tea, a commonly consumed beverage, contains high amounts of polyphenols that can impair protein digestibility, as demonstrated in vitro. There are no human studies examining the inhibitory influence of tea polyphenols (TPP) on high-quality protein digestibility.

**Objective:**

The aim of this study was to determine the effect of black tea on the true indispensable amino acid (IAA) digestibility of whole boiled egg protein, in healthy adult humans, through use of a dual isotope tracer approach.

**Methods:**

The effect of black TPP (4.6 mg/mL, ingested as a beverage with the meal) on ^2^H-labeled whole boiled egg protein, administered with ghee rice and tomato curry, was measured with reference to ^13^C-spirulina protein in healthy Indian adults aged 20–27 y of both sexes with BMI of 22.0 ± 2.8 kg/m^2^. The results were then compared to previously determined whole egg mean IAA digestibility measured by the same method, without black tea, in the same subjects (*n *= 5). To correct for any independent effect of TPP on spirulina protein (used as a standard protein), the true IAA digestibility of ^13^C-spirulina protein was independently measured with reference to a ^2^H-amino acid mixture, with and without co-ingestion of black tea, in 3 of the same subjects.

**Results:**

The true IAA digestibility of whole boiled egg protein significantly decreased by 17% when co-ingested with black tea. However, there was no significant reduction in the true IAA digestibility of spirulina protein when co-ingested with black tea.

**Conclusions:**

TPP protein interactions reduced whole egg digestibility in healthy Indian adults but had minimal effect on spirulina protein digestibility. In populations who are at risk of dietary quality protein inadequacy, the consumption of tea during or after a meal can further increase the risk of inadequacy. This trial was registered at Clinical Trials Registry of India (http://ctri.nic.in) as CTRI/2018/03/012265.

## Introduction

Habitually consumed diets of food-insecure populations, particularly those from low- and middle-income countries, are predominantly plant based, with only a modest increase in animal source foods (ASFs) such as eggs and milk over time (1). In India, diets are mainly cereals, with relatively low-quality proteins because of their low lysine content, and poor digestibility (78% in children) when compared to ASF (87% in children or 92% in adults) (2–4). The proportion of the adult population at risk of quality protein inadequacy is about 24%, nearly doubling in a poor environment because of additional demand and possible adverse effects of impaired intestinal function (2, 5, 6). Plant food matrices reduce protein digestibility through antinutritional factors (7) and a robust cell wall structure, and co-ingestion of beverages such as tea with meals could also show a similar effect on plant or animal source proteins. Tea polyphenol (TPP) protein interactions have been implicated in reducing protein bioavailability in vitro and in rodents (8–11). This inhibitory effect of TPP on protein digestibility could be relevant in populations who consume suboptimal quality protein intakes, and in whom tea is a popular beverage that is consumed with meals (12).

In vivo determination of protein digestion and absorption (digestibility) is now possible in humans through use of the dual stable isotope tracer technique, which was developed recently and applied in adults and children to determine the true indispensable amino acid (IAA) digestibility of different protein sources, such as ASF and plant proteins, relative to a standard protein (spirulina) of known digestibility (3, 4, 13).

This study aimed to determine the effect of TPP from black tea (fully fermented tea leaves) on the digestibility of whole hens’ egg protein, a high-quality protein with no intrinsic polyphenols, using the dual stable isotope tracer technique. However, in this method, the digestibility of the test protein (in this study, ^2^H-labeled whole hens’ egg protein) is measured in comparison to a standard protein of known digestibility (^13^C-labeled spirulina protein); it is possible that TPP from co-ingested black tea could affect the digestibility of both the test and the standard protein, thereby yielding no relative measurable effect. Therefore, 2 experiments were conducted. First, using the dual-tracer method, the effect of co-ingestion of tea on the IAA digestibility of whole hens’ egg protein was measured against a standard protein (^13^C-labeled spirulina protein), in subjects in whom whole hens’ egg protein digestibility, without tea, had earlier been measured (4) by the same method. Second, because the true IAA digestibility of the standard protein used in the first experiment (^13^C-labeled spirulina protein) could also be affected by TPP, the digestibility of ^13^C-spirulina protein was measured with and without the same amount of co-ingested black tea (as in the first experiment), in the same subjects, relative to a standard of a ^2^H-labeled crystalline IAA mixture.

## Methods

Subjects in whom whole boiled egg protein digestibility had earlier been determined with reference to a spirulina protein standard (4) were approached, because their data on true IAA digestibility of egg protein without tea could be used as control for this study. The present study was conducted 2 mo later, and the subjects reported no gastrointestinal symptoms or illnesses during the intervening period. Five subjects (*n *= 3 women and *n *= 2 men) consented to be studied again for determining whole egg protein digestibility against the same standard spirulina protein, when ingested with black tea. Because the independent effect of black tea on spirulina protein (used as a standard protein in the egg protein digestibility assessment) also needed to be characterized, 3 (*n *= 1 woman and *n *= 2 men) of the 5 subjects consented to additional measurements of spirulina protein digestibility referenced to a standard crystalline ^2^H-amino acid mixture, with and without the ingestion of black tea.

The subjects were within the normal BMI range (18.5–25 kg/m^2^), aged between 20 and 27 y, had no food allergies, and did not smoke. They had no serious illness 3 mo before the study, were not on antibiotics within 4 wk before the study, and had not consumed alcohol in the previous 24 h. Details of subject screening and enrolment are provided in **Supplemental Figure 1**. The study was approved by the institutional ethical review board and all subjects provided informed written consent.

To determine the effect of black tea polyphenols on spirulina protein digestibility, 2 sequential test meals containing [U-^13^C]-labeled spirulina protein (test protein, Cambridge Isotope Laboratories) and a [U-^2^H]-labeled amino acid mixture (standard protein, Cambridge Isotope Laboratories) were administered with and without black tea. The composition of the [U-^2^H]-labeled amino acid mixture is given in **Supplemental Table 1**. For the whole egg protein digestibility measurement, [U-^2^H]-labeled whole boiled egg protein (test protein) and [U-^13^C]-spirulina (standard protein, with digestibility measured as above, with and without black tea), were administered with and without the same quantity of black tea. The ^2^H-intrinsically labeled hens’ eggs were produced as described earlier (4). Briefly, 2 hens (layers) were orally dosed with 12 mg/d of [U-^2^H]-labeled crystalline amino acid mixture and eggs were collected. The labeled eggs were then boiled, shelled, and stored immediately at −80°C. For the experiment, the eggs were thawed at 4°C overnight, minced, and added to the curry.

A culturally acceptable meal consisting of ghee rice and tomato curry was used as the base meal for all experiments. The protein content of the meals that had no egg protein (as in the spirulina digestibility experiment) was kept constant by adding unlabeled chickpeas as the protein source. The unlabeled chickpeas were soaked overnight for 12 h, pressure cooked on the morning of the experiment, and added to the curry as needed. The test meals provided one-third of the daily energy and protein requirements for adults, of which the egg or chickpea contributed two-thirds of the protein in the meal. The nutrient compositions of the test meals are provided in **Table 1**. The black tea was prepared by adding 15 g of black tea powder (Brooke Bond, Red Label) to 240 g of boiling water for 10 min. The test meal and black tea were divided into aliquots (see below) for plateau feeding and were warmed in a microwave for 10 s before they were given to the subjects.

**TABLE 1:** Nutrient composition of the standardized egg and spirulina test meals consumed by Indian adults with and without black tea^1^

	Egg	Egg + tea	Spirulina	Spirulina + tea
Energy, kcal/meal	691 ± 44.6	697 ± 47.7	766 ± 44.5	767 ± 45.7
Protein, g/meal	21.5 ± 0.5	21.7 ± 0.9	19.8 ± 0.6	19.9 ± 0.6
Fat, g/meal	29.7 ± 0.9	29.95 ± 1.1	24.6 ± 0.2	24.6 ± 0.3
Carbohydrate, g/meal	83.7 ± 8.7	84.4 ± 8.9	115.9 ± 9.8	116.1 ± 10.0
PE ratio	12.5 ± 0.6	12.5 ± 0.6	10.4 ± 0.3	10.4 ± 0.3

1 Values are means ± SDs, *n *= 5 for egg ± tea meal, *n *= 3 for spirulina ± tea meal, paired studies in subjects for egg and egg + tea and spirulina and spirulina + tea. The subjects in spirulina ± tea were a subset from the egg ± tea study. The nutrient composition of the egg meal is the mean ± SD of *n *= 5 subjects from a previously published whole boiled egg study (4). The standardized meal consisted of a culturally acceptable tomato curry and ghee rice including the test protein. PE, protein energy ratio.

On the day of the digestibility measurement, subjects reported at 0630 to the metabolic unit after an overnight fast of 10 h. The experiment started at 0700 and continued for the next 8 h. The subjects were restricted to minimal physical activity during the experiment. A primed plateau feeding protocol was initiated, in which the whole meal with the isotopes was divided into mini-meals and fed at hourly intervals. The cooked test meal was portioned into 11 parts, each part constituting 1 mini-meal and the black tea was portioned into 10 parts, each part consisting of 1 mini-cup, which on an average was 13 g. A priming meal was fed (consisting of 3 mini-meals) along with ^13^C-bicarbonate as a priming dose for the bicarbonate pool (4 mg/kg, Cambridge Isotope Laboratories, >99% purity), with or without tea (priming dose of 3 mini-cups). This was followed by hourly single mini-meals, with or without a mini-cup of tea, depending on the experiment, for the next 7 h. One of the mini-meal portions was retained for isotopic analysis. The test meals and black tea were tested for their total polyphenol content (TUV SUD) using the Folin Ciocalteu (14) method.

A basal blood sample was collected after securing an indwelling venous catheter (Jelco 22 G, Medex Medical Ltd) followed by half hourly samples from the fifth to eighth hours (a total of 8 blood samples were collected), representing the plateau fed-state period (13). At each time point 4 mL of blood was withdrawn. Whole blood was transferred into EDTA-coated evacuated tubes (Becton Dickenson) to separate plasma in a refrigerated centrifuge, which was then aliquoted and stored at −80°C until analysis. Breath samples were collected using 10 mL evacuated plain glass tubes (Becton Dickenson) at baseline, followed by hourly collections for the experimental duration. These samples were stored at room temperature until analysis.

Plasma samples were deproteinized, and amino acids were collected by cation exchange and derivatized to their ethoxycarbonyl ethyl esters. The [^13^C] and [^2^H] isotopic enrichments of the IAA were analyzed by liquid chromatography with tandem mass spectrometry (6495 QQQ with i-Funnel technology, Agilent), as explained in detail earlier (13). Whole meal samples underwent gas phase acid hydrolysis before measurement of [^13^C] and [^2^H] isotopic abundance of the IAA (except for tryptophan) in the meal. Baseline meal isotopic abundance was measured in similar test meals containing unlabeled whole boiled egg and spirulina. The [^13^C] and [^2^H] enrichments of IAA were expressed as parts per million excess (ppme) over the baseline. Breath samples were analyzed for ^13^CO_2_ abundance with use of isotope ratio mass spectrometry (Delta V Advantage, Thermo Fisher Scientific Inc.).

The true IAA digestibility (%) of spirulina protein with or without black tea was calculated as:

(1) \begin{eqnarray*}
&&\big[ {{\rm{Plasma}}{\,^{13}}{\rm{C - IAA}}\big( {{\rm{ppme}}} \big)/{\rm{Meal}}{\,^{13}}{\rm{C - IAA}}\big( {{\rm{ppme}}} \big)} \big]\big/ \nonumber \\ 
&&\big[ {{\rm{Plasma}}{\,^2}{\rm{H - IAA}}\big( {{\rm{ppme}}} \big)/{\rm{Meal}}{\,^2}{\rm{H - IAA}}\big( {{\rm{ppme}}} \big)} \big] \times 100
\end{eqnarray*}

The true IAA digestibility (%) of whole boiled egg protein without black tea was taken from the previous study (4), and was recalculated as:

(2) \begin{eqnarray*}
&& \big[ {{\rm{Plasma}}{\,^2}{\rm{H - IAA}}\big( {{\rm{ppme}}} \big)/{\rm{Meal}}{\,^2}{\rm{H - IAA}}\big( {{\rm{ppme}}} \big)} \big]\big/ \nonumber \\
&& \big[ {{\rm{Plasma}}{\,^{13}}{\rm{C - IAA}}\big( {{\rm{ppme}}} \big)/{\rm{Meal}}{\,^{13}}{\rm{C - IAA}}\big( {{\rm{ppme}}} \big)} \big] \nonumber \\
&&\quad \times 100 \times \left( {{\rm{Di}}{{\rm{g}}_{{\rm{Std}}}}/100} \right)
\end{eqnarray*}

where, Dig_Std_ = mean true IAA digestibility of spirulina protein determined in the present study with reference to crystalline IAA (as above), without the ingestion of black tea. Because these paired spirulina IAA digestibility values were available for only 3 subjects and digestibility values of spirulina matched with that of an earlier study in subjects with similar characteristics (13), the mean of these values was used for the other 2 subjects.

The true IAA digestibility (%) of whole boiled egg protein co-ingested with black tea was calculated as:

(3) \begin{eqnarray*}
&& \left[ {{\rm{Plasma}}{\,^2}{\rm{H - IAA}}\left( {{\rm{ppme}}} \right)/{\rm{Meal}}{\,^2}{\rm{H - IAA}}\left( {{\rm{ppme}}} \right)} \right]\big/ \nonumber \\
&& \left[ {{\rm{Plasma}}{\,^{13}}{\rm{C - IAA}}\left( {{\rm{ppme}}} \right)/{\rm{Meal}}{\,^{13}}{\rm{C - IAA}}\left( {{\rm{ppme}}} \right)} \right] \nonumber \\
&& \quad \times 100 \times \left( {{\rm{Di}}{{\rm{g}}_{{\rm{Std - TPP}}}}/100} \right)
\end{eqnarray*}

where, Dig_Std-TPP_ = mean true IAA digestibility of spirulina protein determined in the present study with reference to crystalline amino acid mixture, when black tea was co-ingested (as above). As noted earlier, because paired Dig_Std-TPP_ values were available for only 3 consenting subjects, the mean Dig_Std-TPP_ was used for the other 2 subjects.

The number of subjects needed to evaluate differences in egg protein digestibility between the black tea conditions was calculated using an observed difference of 6% in mean digestibility between chicken meat and lyophilized egg-white protein from an earlier study (4). Five subjects were required to detect a 12% significant difference (doubling the difference previously observed) in digestibility between egg protein with and without tea, at a 5% level of significance, with a power of 80%. Because the mean and median of the mean true digestibility and the individual IAA digestibility values were similar and the SDs were low (< half of the mean), the data were assumed to be normally distributed. Paired *t* tests were performed to evaluate the difference between the mean and individual IAA true digestibilities of the egg with and without black tea (*n *= 5); and spirulina with and without black tea (*n *= 3). For all the comparisons, *P *< 0.05 was considered to be significant. All calculations were performed on SPPS Statistics, version 17.0 (IBM).

## Results

The demographics and clinical assessment of the subjects in both experiments is provided in **Table 2**. The TPP content of the test meals approximately doubled when black tea was added; for the spirulina protein digestibility meal, these were 0.22% and 0.12% (wt:wt) with and without black tea, respectively, and for the whole boiled egg protein digestibility meal, these were 0.29% and 0.15% (wt:wt), respectively. The ^2^H- and ^13^C-IAA enrichments (ppme) of the test meals are given in **Supplemental Table 2**. The ^2^H- and ^13^C-plasma enrichments of each IAA at plateau (from the fifth to the eighth hour of the experimental protocol) are shown in **Supplemental Figure 2**, and the mean ^2^H- and ^13^C-IAA plasma enrichments at plateau are given in **Supplemental Table 3**. The mean interindividual CV of the ^2^H- and ^13^C-plasma IAA enrichments at plateau were 24% and 19%, respectively. These values ranged from 19% for methionine to 33% for lysine for specific ^2^H-plasma IAA enrichments, and from 17% for phenylalanine and 23% for valine for specific ^13^C-plasma IAA enrichments. The breath ^13^CO_2_ enrichment reached a similar plateau value after 5 h in all 4 experiments (2 for egg protein and 2 for spirulina protein digestibility, with and without black tea) indicating a similar oxidative disposal of [U-^13^C]-labeled spirulina (**Supplemental Figure 3**) (4).

**TABLE 2:** Demographic characteristics and anthropometry of Indian adults who participated in egg and spirulina with and without black tea experiments^1^

Variables	Egg ± tea	Spirulina ± tea
Age, y	23.6 ± 2.5	23.7 ± 3.5
Weight, kg	55.3 ± 3.6	55.3 ± 2.2
Height, m	1.6 ± 0.1	1.6 ± 0.1
BMI, kg/m^2^	22.0 ± 2.8	21.0 ± 1.8
Hemoglobin, g/dL	12.7 ± 1.4	13.2 ± 1.6

1 Values are means ± SDs, *n* = 5 for egg ± tea (*n *= 3 women and *n *= 2 men) and *n* = 3 for spirulina ± tea experiment (*n *= 1 woman and *n *= 2 men), paired studies in subjects for egg and egg + tea; and spirulina and spirulina + tea.

2 The subjects in spirulina ± tea were a subset from egg ± tea study.

**FIGURE 1:**
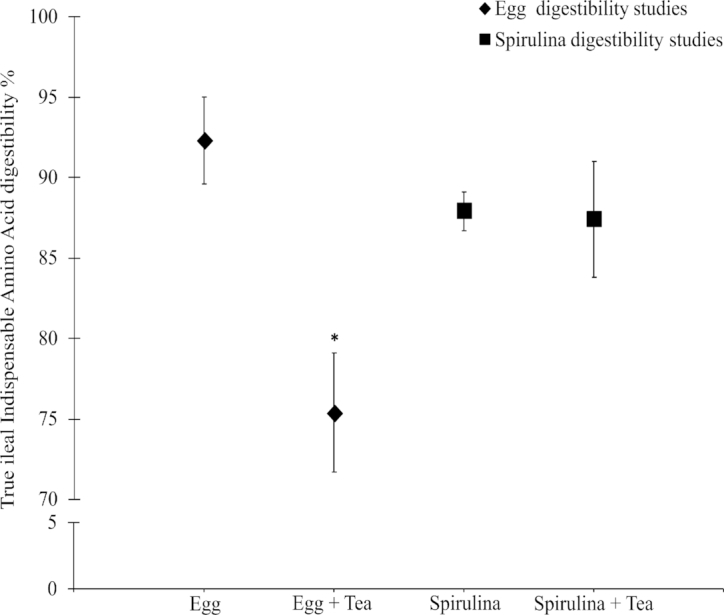
True indispensable amino acid digestibility of whole egg and spirulina protein consumed without and with black tea in Indian adults. Values are means ± SDs, *n* = 5 (egg) or 3 (sprirulina). Paired *t* tests were performed within egg and spirulina studies. *Different from egg, *P *< 0.001.

The true mean or individual IAA digestibility of spirulina protein ingested without and with black tea did not differ significantly (*P *= 0.803); the differences in individual IAA digestibility were minimal, in the range of 0.3% (valine) to 7.4% (methionine) (**Table 3**). However, there was a significant effect of black tea on the mean true IAA digestibility of whole boiled egg when co-ingested without and with black tea, resulting in a significant reduction of 17% (*P *< 0.001, **Figure 1**). A similar significant reduction was noted for the individual true IAA digestibility of whole boiled egg protein, when ingested with black tea (*P *< 0.05 for all IAA, Table 3). This difference in IAA digestibility ranged from 12% lower for leucine to 22% lower for lysine. Because the calculation of digestibility involved a 2-step measurement, meaning that the digestibility of the standard was measured separate to the measurement of egg digestibility, the overall variance was estimated as the variance of the product of egg and standard protein digestibilities. The variability remained the same for egg without tea (2.7%), whereas it increased to 5% for egg with tea.

**TABLE 3:** True IAA digestibility values for hens’ whole boiled egg and spirulina protein with and without black tea in Indian adults^1^

	True IAA digestibility, %					
	Egg *n *= 5	Egg + tea^2^*n *= 5	*P* value	Spirulina *n *= 3	Spirulina + tea^2^*n *= 3	*P* value
Methionine	87.3 ± 7.3	71.9 ± 10.3	0.024	86.2 ± 3.4	93.6 ± 8.6	0.169
Phenylalanine	93.3 ± 2.4	76.8 ± 10.9	0.041	93.1 ± 2.6	88.2 ± 18.0	0.718
Threonine	95.8 ± 1.4	75.0 ± 9.6	0.007	82.3 ± 2.7	83.5 ± 14.2	0.890
Lysine	102.2 ± 6.7	80.6 ± 6.0	<0.001	89.2 ± 1.2	87.7 ± 6.2	0.654
Leucine	88.5 ± 3.0	76.6 ± 2.4	0.001	86.7 ± 1.5	85.0 ± 3.2	0.549
Iso-leucine	87.2 ± 3.3	70.2 ± 3.9	0.003	85.4 ± 3.1	82.0 ± 5.2	0.531
Valine	92.1 ± 6.3	74.0 ± 3.3	0.010	92.4 ± 3.0	92.1 ± 5.5	0.858

1 Values are means ± SDs; paired studies. The subjects in spirulina ± tea were a subset from the egg ± tea study. IAA, indispensable amino acid.

2 Paired *t* tests were performed within egg and spirulina studies.

## Discussion

This study determined the inhibitory effect of black TPP on the true IAA digestibility of whole boiled egg protein, using the dual isotope tracer technique. The approach involves comparison of 2 differentially labeled proteins, a test (whole egg) and a standard (spirulina) protein, which are ingested together. However, in this method, if the digestibility of both proteins is equally affected by black tea, the specific TPP effect on egg protein digestibility will not be discernible. Therefore, the present study independently determined the effect of black tea on spirulina protein, in comparison to a standard of labeled crystalline amino acid mixture that does not require digestion. As observed, co-ingestion of black tea resulted in a significant decrease of 17% in the mean true IAA digestibility of whole egg protein in comparison to spirulina protein, but there was no specific significant effect of black tea on spirulina protein.

The effect of TPP on protein digestibility appears to vary depending on the source of the protein. This has been studied extensively in vitro for beverages such as black tea, green tea, and coffee on varying protein sources; however, there are limited studies in animal models and none in humans ( 11, 15). Among the in vitro studies of the effect of black TPP on egg protein, 1 found a dose-dependent decrease in digestibility of spray dried egg yolk protein. The details of the assay are not available, but it appears that with use of a similar black tea content to that used in the present study, the reduction in digestibility of egg yolk protein was 32% (8). In another in vitro study, the addition of quercetin, a pure extract of black TPP (at 2 μM, which compares to 4 times the concentration given through black tea in the present study), decreased the enzymatic (trypsin and chymotrypsin) digestion of egg white and yolk proteins by 6% and 10%, respectively (9). Methodological differences, accessibility of the proteins within the food matrix, type and concentration of digestive proteases and substrates used, and duration of the reaction, could explain the variation in the reduction observed between the studies.

TPP protein interactions have been studied in great detail in vitro, and depend on the polyphenol structure and molecular size, the amino acid composition and secondary or tertiary structure of the protein, and the pH (which influences protein folding); these may be either covalent or noncovalent interactions (hydrogen bonds or hydrophobic interactions) (10, 16). TPP interacts both with the substrates (dietary protein) and the proteases that hydrolyze them (15, 17). The binding of TPP to proteases can alter the active configuration of the enzyme by destabilizing the orientation of the active site, or by allosteric inhibition (15). Black TPP extracts, which are rich in high molecular weight polyphenols, strongly inhibited trypsin activity at 0.01 mg/mL, a lower concentration than used in the present study (4.6 mg/mL of black TPPs) (18). Among the amino acids, the aromatic, amino, and thiol R-groups are prone to interactions with TPP (16, 19), rendering them inaccessible to protease activity. In particular, TPP interactions with specific egg proteins (ovalbumin and lysozyme) were related to secondary structural changes of the proteins that promoted their digestion by pepsin in acidic pH but inhibited their digestion by bovine pancreatic extract in alkaline conditions (10).

In this study, spirulina protein digestibility was not significantly affected by TPP. This could be because of intrinsic polyphenols or molecules that bind to proteins and reduce the effect of extrinsic polyphenols; an example is the presence of phycobilisomes (phycobiliprotein-chromophore complexes). Multiple chromophores are known to bind to phycobiliproteins mainly through sulfhydryl groups of cysteine and through noncovalent interactions with surrounding amino acids. Because the amino acid side chains are already engaged, TPP associations with spirulina proteins may be minimal (20). Similarly, in other protein foods, the presence of varied amounts of intrinsic polyphenols or molecules that bind to proteins might influence the effect of extrinsic polyphenols on protein digestibility. For example, the fecal IAA digestibility of barley and soya in rats, determined by fecal nitrogen balance, reduced by only about 5% and 3% when co-administered with a black tea extract (11).

The matrix in which the protein is consumed affects its digestibility. Antinutritional factors such as protease inhibitors, polyphenols, tannins, and phytic acid in the food matrix lower protein digestibility (7), affecting plant sources more than ASF (4, 13). However, as this study shows, the concomitant consumption of an ASF (egg) with tea, reduces its protein digestibility substantially. Beverages rich in polyphenols, such as tea and coffee, are generally taken after meals, especially following breakfast (12). In India, the mean per capita daily intake of tea is 11 g, ranging from 2 to 25 g between different Indian states (21). The consumption of tea has been increasing over time, with a predicted increase of 3% per annum (22). Because of its affordability, there is no distinction in consumption of tea between socioeconomic groups. Although multiple health benefits are ascribed to the antioxidant and anti-inflammatory properties of TPP, deleterious effects on nutrient bioavailability also exist. The amount of TPP given in this study during the plateau feeding protocol was equivalent to 100 mL of 6 cups of black tea, 2 cups of coffee, and 10 cups of green tea (23). However, this can vary, as the TPP content of black tea infusions has been reported to range from 13 to 100 mg gallic-acid equivalents/g of dry matter, depending on the type of black tea used and the infusion or brewing time (24, 25).

In the Indian population, where diets are cereal-based, there is 24% (urban and rural combined) risk of dietary quality protein inadequacy (2). With additional demand resulting from consumption of tea or coffee (assuming a 5% reduction in digestibility if 1 cup of tea or coffee was consumed with 1 of the major meals), along with the extra demand of a poor environment, the risk, based on survey data (21) and assumptions reported in Minocha et al. (2), could increase to 43%. This is particularly important in vulnerable populations (growing children, women of reproductive age, and the elderly), with the potential to adversely impact growth and functionality. Equally, the effect may be lower in plant protein sources such as legumes, that are otherwise rich in polyphenols, but still may be significant when legumes are the principal source of high-quality protein.

The strength of this study is the design that allowed for paired individual determination of the effect of TPP on egg protein digestibility in vivo and the use of a relatively minimally invasive method to determine IAA digestibility when compared to nasogastric intubation or ileostomy. However, since dual isotope tracer technique has been recently developed, there is still need for validations against standard methods that directly measure IAA digestibility at the ileum. Limitations of this method include the assumption that amino acids are completely absorbed and amino acids from both test and the standard protein undergo identical splanchnic extraction. A limitation of the design is the use of control data on whole boiled egg (without black tea) digestibility from an earlier experiment, even though the same subjects were studied, and the repeat measurement with co-ingestion of tea was made within a period of 2 mo from the earlier experiment. In addition, the subjects were healthy to begin with, and remained apparently healthy in the intervening period, with no gastrointestinal symptoms. Another limitation was that because the variability of individual IAA digestibility increased on co-ingestion of TPP with spirulina, the possibility of an inhibitory effect of TPP on spirulina cannot be completely ruled out. Further, it was desirable that in this set of successive experiments on the same subjects, a subject-specific spirulina digestibility (without tea) would be used as the standard protein digestibility in the first experiment, in which egg protein digestibility was measured relative to the spirulina protein as the standard. However, spirulina protein digestibility was not measured in 2 of the 5 subjects because of their unavailability; therefore, the mean measured spirulina IAA digestibility was used as the standard protein digestibility value for these 2 subjects. This is reasonable, because earlier measurements of spirulina digestibility against a standard of crystalline ^2^H-IAA had shown low interindividual variability, and in the present study, interindividual variation in IAA digestibility in the 3 subjects was low (ranging from 1.2% for lysine to 3.4% for methionine). Finally, the measured effect of tea requires further investigation when ingested as an infusion with milk, because tea is culturally drunk with milk in India (12). Milk protein has also been shown to interact with chocolate polyphenols, reducing their bioavailability in vivo (26). Therefore, the reductive effect of TPP on digestibility of co-ingested dietary protein may be different when consumed as tea with milk, but this needs to be tested with labeled milk protein.

In conclusion, co-ingestion of high-quality egg protein with black tea is detrimental for its true IAA digestibility. In populations subsisting on a cereal diet with small amounts of high-quality protein foods, this effect could further contribute to the risk of protein inadequacy, but the complex effects of a mixed food matrix, including milk, need to be evaluated. In addition, the detrimental effect is not uniform, and could be minimal with foods that have other molecules inherently binding to their proteins.

## Supplementary Material

nxz091_Supplemental_Files

## Notes

Supported by The Wellcome Trust/DBT India Alliance through the Margdarshi fellowship to AVK.

Author disclosures: SK, NS, AV, TP, SD, and AVK, no conflicts of interest.

SK and NS contributed equally to this article and share first authorship.

Supplemental Figures 1–3 and Supplemental Tables 1–3 are available from the “Supplementary data” link in the online posting of the article and from the same link in the online table of contents at https://academic.oup.com/jn/.

Abbreviations used: ASF, animal source food; IAA, indispensable amino acid; ppme, parts per million excess; TPP, tea polyphenol.

## References

[1] Kearney J Food consumption trends and drivers. Philos Trans R Soc Lond B Biol Sci. 2010;365(1554):2793–807. 20713385 10.1098/rstb.2010.0149 PMC2935122

[2] Minocha S, Thomas T, Kurpad AV Dietary protein and the health-nutrition-agriculture connection in India. J Nutr. 2017;147(7):1243–50. 28515162 10.3945/jn.116.243980

[3] Shivakumar N, Kashyap S, Kishore S, Thomas T, Varkey A, Devi S, Preston T, Jahoor F, Sheshshayee MS, Kurpad AV Protein-quality evaluation of complementary foods in Indian children. Am J Clin Nutr. 2019;; pii: nqy265. doi: 10.1093/ajcn/nqy265. [Epub ahead of print]. 10.1093/ajcn/nqy265 PMC6499502 30920607

[4] Kashyap S, Shivakumar N, Varkey A, Duraisamy R, Thomas T, Preston T, Devi S, Kurpad AV Ileal digestibility of intrinsically labeled hen's egg and meat protein determined with the dual stable isotope tracer method in Indian adults. Am J Clin Nutr. 2018;108(5):980–7. 30272112 10.1093/ajcn/nqy178 PMC6250983

[5] Crane RJ, Jones KD, Berkley JA Environmental enteric dysfunction: an overview. Food Nutr Bull. 2015;36(Suppl 1):S76–87. 25902619 10.1177/15648265150361S113 PMC4472379

[6] Kurpad AV, Regan MM, Nazareth D, Nagaraj S, Gnanou J, Young VR Intestinal parasites increase the dietary lysine requirement in chronically undernourished adult Indian subjects. Am J Clin Nutr. 2003;78:1145–51. 14668277 10.1093/ajcn/78.6.1145

[7] Gilani GS, Cockell KA, Sepehr E Effects of antinutritional factors on protein digestibility and amino acid availability in foods. J AOAC Int. 2005;88(3):967–87. 16001874

[8] Yenrina R, Permata DA, Rasjmida D, Tayandi R In vitro protein digestibility and physical properties of instant teh talua dried by spray dryer. Int J Adv Sci Eng Info Tech. 2016;6(1):84–7.

[9] Hassan MS. Egg protein interactions with phenolic compounds: effect on protein properties (Doctoral dissertation, McGill University).

[10] Shen F, Niu F, Li J, Su Y, Liu Y, Yang Y Interactions between tea polyphenol and two kinds of typical egg white proteins—ovalbumin and lysozyme: effect on the gastrointestinal digestion of both proteins in vitro. Food Res Int. 2014;59:100–7.

[11] Eggum BO, Pedersen B, Jacobsen I The influence of dietary tea, coffee and cocoa on protein and energy utilization of soya-bean meal and barley in rats. Br J Nutr. 1983;50(2):197–205. 6684477 10.1079/bjn19830089

[12] Tea Board. Executive Summary on Domestic Consumption of Tea in India. 2007.

[13] Devi S, Varkey A, Sheshshayee MS, Preston T, Kurpad AV Measurement of protein digestibility in humans by a dual-tracer method. Am J Clin Nutr. 2018;107(6):984–91. 29771297 10.1093/ajcn/nqy062 PMC6179135

[14] ISO ISO 14502-1: 2005, Determination of substances characteristic of green and black tea—Part 1: Content of total polyphenols in tea- colorimetric method using Folin-Ciocalteu reagent. 2005.

[15] Cirkovic Velickovic TD, Stanic‐Vucinic DJ The role of dietary phenolic compounds in protein digestion and processing technologies to improve their antinutritive properties. Comp Rev Food Sci Food Safety. 2018;17(1):82–103. 10.1111/1541-4337.12320 33350063

[16] Kroll J, Rawel HM, Rohn S Reactions of plant phenolics with food proteins and enzymes under special consideration of covalent bonds. Food Sci Tech Res. 2003;9(3):205–18.

[17] He Q, Lv Y, Yao K Effects of tea polyphenols on the activities of α-amylase, pepsin, trypsin and lipase. Food Chem. 2007;101(3):1178–82.

[18] Łoś J, Podsędek A. Tannins from different foodstuffs as trypsin inhibitors. Pol J Food Nutr Sci. 2004;13/54(1):51–5.

[19] Rawel HM, Meidtner K, Kroll J Binding of selected phenolic compounds to proteins. J Agri Food Chem. 2005;53(10):4228–35. 10.1021/jf0480290 15884865

[20] Padyana AK, Bhat VB, Madyastha KM, Rajashankar KR, Ramakumar S Crystal structure of a light-harvesting protein C-phycocyanin from Spirulina platensis. Biochem Biophys Res Commun. 2001;282(4):893–8. 11352634 10.1006/bbrc.2001.4663

[21] National Sample Survey Office. Level and pattern of consumption expenditure, 2011–12. NSS 68th round [Internet]. NSS Report No.: 555 New Delhi (India): Government of India, National Sample Survey Office; 2014; [cited 2017 Feb 2]. Available from: http://www.indiaenvironmentportal.org.in/files/file/Level%20and%20Pattern%20of%20Consumer%20Expenditure_0.pdf.

[22] Chang K World tea production and trade: current and future development. [Internet]. Rome: A publication by the Food and Agricultural Organization of the United Nations; Available from: www.fao.org 2015.

[23] Rothwell JA, Perez-Jimenez J, Neveu V, Medina-Remon A, M'Hiri N, García-Lobato P, Manach C, Knox C, Eisner R, Wishart DS et al. Phenol-Explorer 3.0: a major update of the Phenol-Explorer database to incorporate data on the effects of food processing on polyphenol content. Database. 2013;2013:bat070. 24103452 10.1093/database/bat070 PMC3792339

[24] Nikniaz Z, Mahdavi R, Ghaemmaghami SJ, Yagin NL, Nikniaz L Effect of different brewing times on antioxidant activity and polyphenol content of loosely packed and bagged black teas (Camellia sinensis L.). Avicenna J Phytomed. 2016;6(3):313. 27462554 PMC4930538

[25] McAlpine MD, Ward WE Influence of steep time on polyphenol content and antioxidant capacity of black, green, rooibos, and herbal teas. Beverages. 2016;2(3):17.

[26] Serafini M, Bugianesi R, Maiani G, Valtuena S, De Santis S, Crozier A Plasma antioxidants from chocolate. Nature. 2003;424(6952):1013. 12944955 10.1038/4241013a

