# Supplementary material for: Co-ingestion of Black Tea Reduces the Indispensable Amino Acid Digestibility of Hens’ Egg in Indian Adults
Source: J Nutr. 2019 May 25;149(8):1363–8. doi: 10.1093/jn/nxz091 (PMC6682489; doi:10.1093/jn/nxz091)
Supplement: nxz091_Supplemental_Files [file nxz091_supplemental_files.zip › Supplemental Figure 1.pdf]

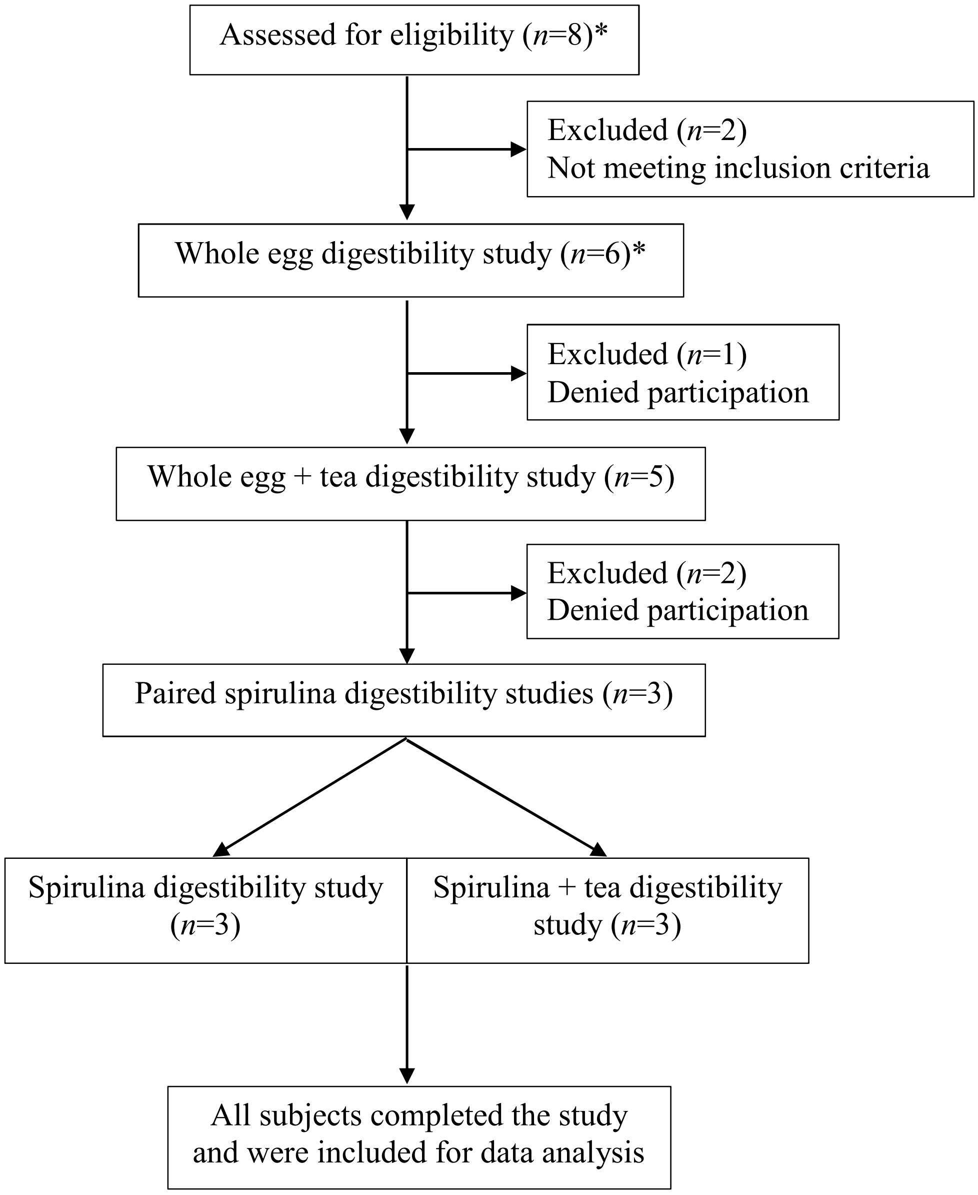

Supplemental Figure 1. Subject screening and enrolment details for egg and spirulina digestibility studies (*previously published)

* Kashyap S, Shivakumar N, Varkey A, Duraisamy R, Thomas T, Preston T, Devi S, Kurpad AV. Ileal digestibility of intrinsically labeled hen's egg and meat protein determined with the dual stable isotope tracer method in Indian adults. Am J Clin Nutr. 2018 Oct 1;108(5):980-7.
