# Supplementary material for: Co-ingestion of Black Tea Reduces the Indispensable Amino Acid Digestibility of Hens’ Egg in Indian Adults
Source: J Nutr. 2019 May 25;149(8):1363–8. doi: 10.1093/jn/nxz091 (PMC6682489; doi:10.1093/jn/nxz091)
Supplement: nxz091_Supplemental_Files [file nxz091_supplemental_files.zip › Supplemental Figure 2.pdf]

Supplementary data

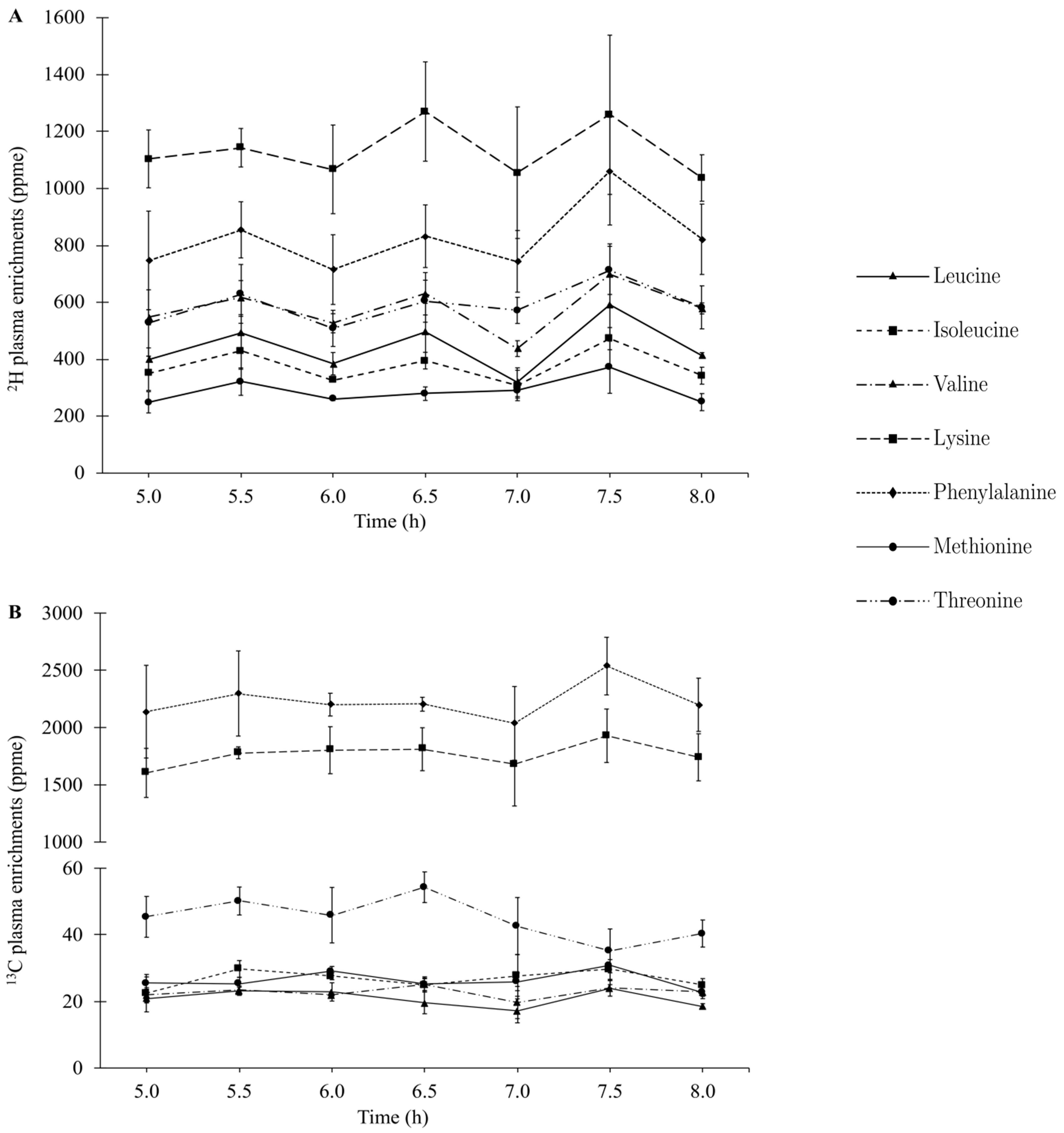

Supplementary data

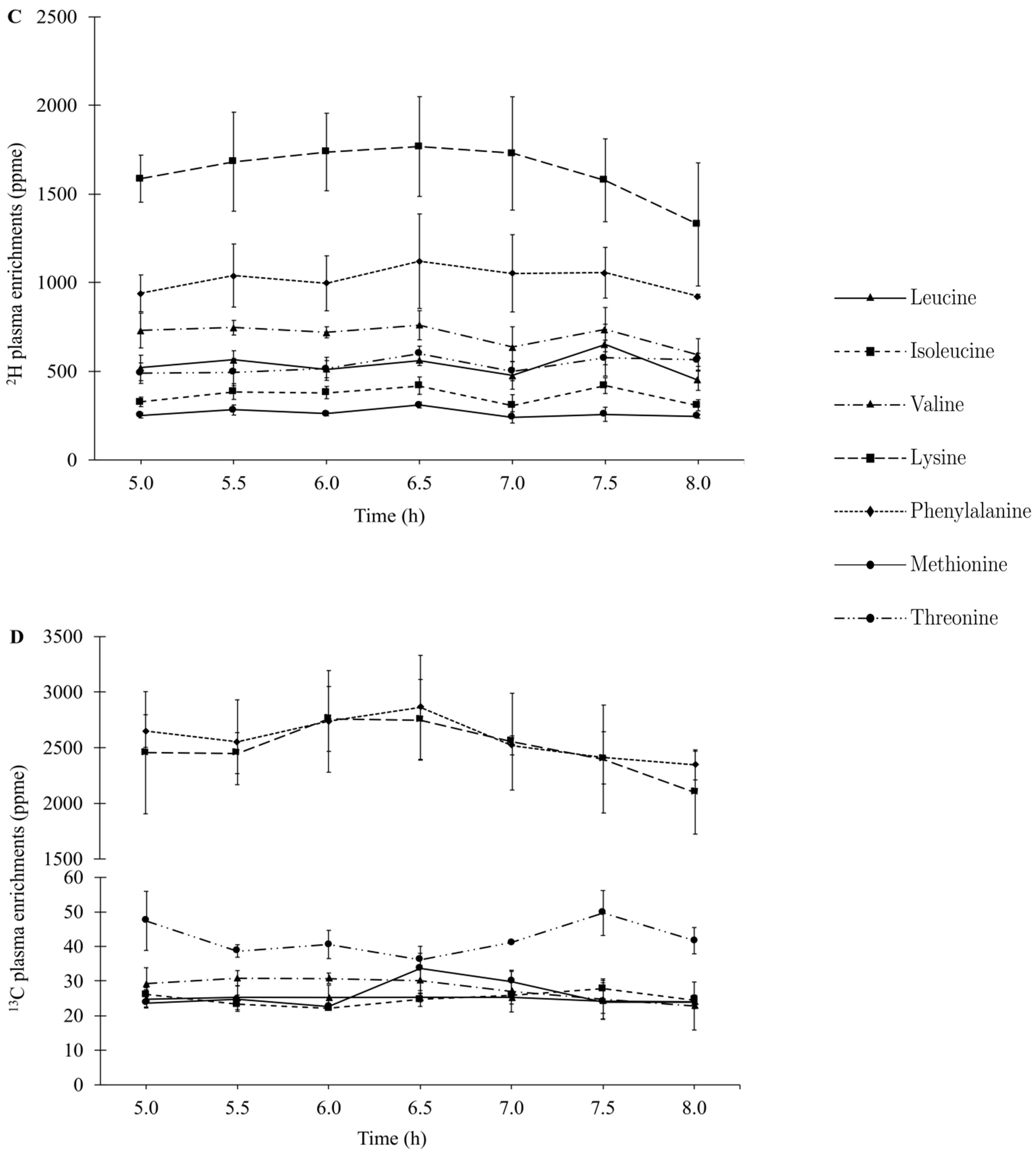

Supplementary data

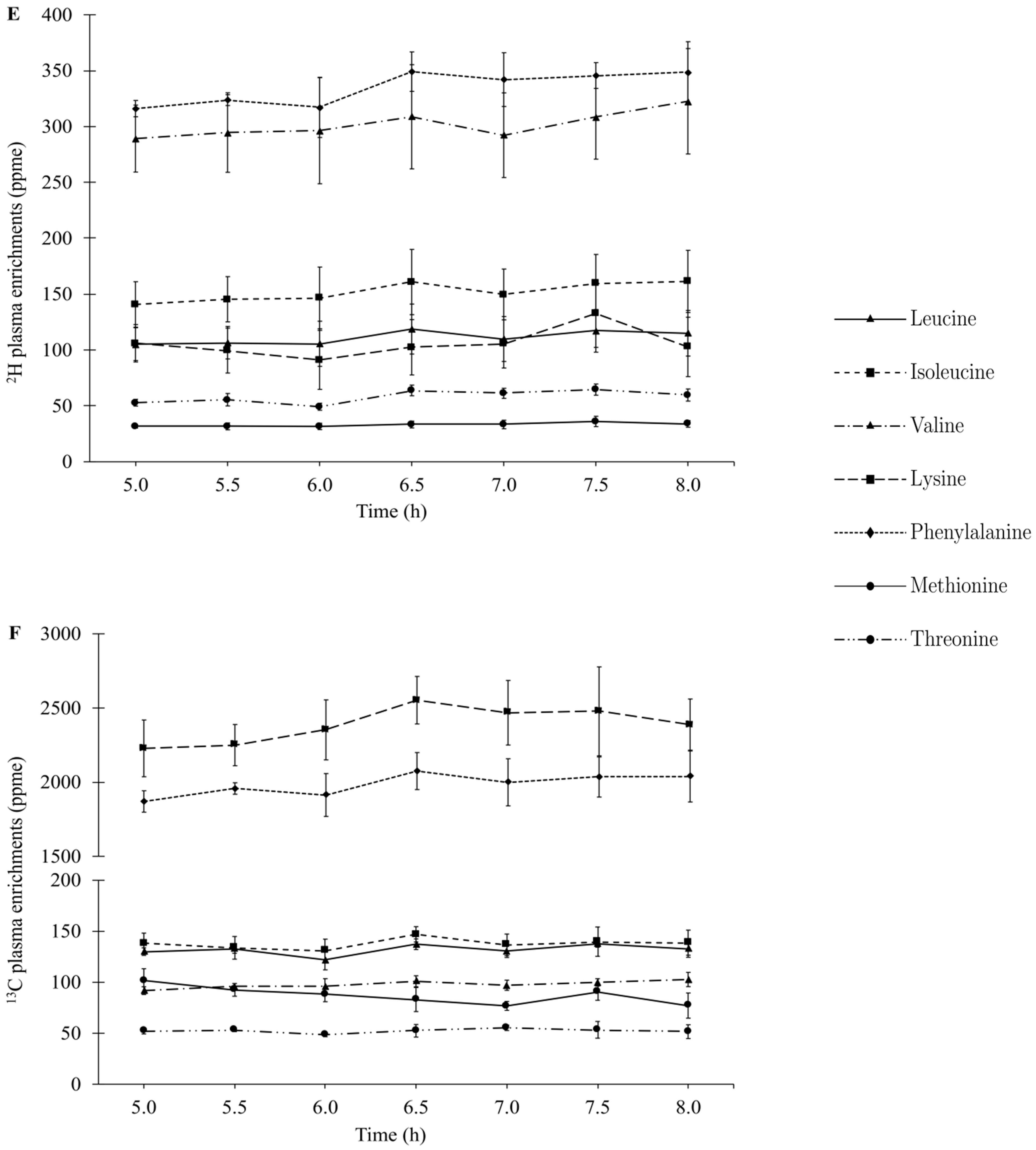

Supplemental Figure 2. Plasma enrichment of IAA after consumption of different test protein meals in healthy Indian adults A) Plasma appearance of ^{2}H and B) ^{13}C isotopic enrichments of IAA (ppme) at plateau after consumption of spirulina without tea meal (*n*=3); C) Plasma appearance of ^{2}H and D) ^{13}C isotopic enrichments of IAA (ppme) at plateau after consumption of spirulina + tea meal (*n*=3) E) Plasma appearance of ^{2}H and F) ^{13}C isotopic enrichments of IAA (ppme) at plateau after consumption of egg + tea meal (*n*=5); mean ± SE

is represented.
