# Supplementary material for: Co-ingestion of Black Tea Reduces the Indispensable Amino Acid Digestibility of Hens’ Egg in Indian Adults
Source: J Nutr. 2019 May 25;149(8):1363–8. doi: 10.1093/jn/nxz091 (PMC6682489; doi:10.1093/jn/nxz091)
Supplement: nxz091_Supplemental_Files [file nxz091_supplemental_files.zip › Supplemental Figure 3.pdf]

Supplementary data

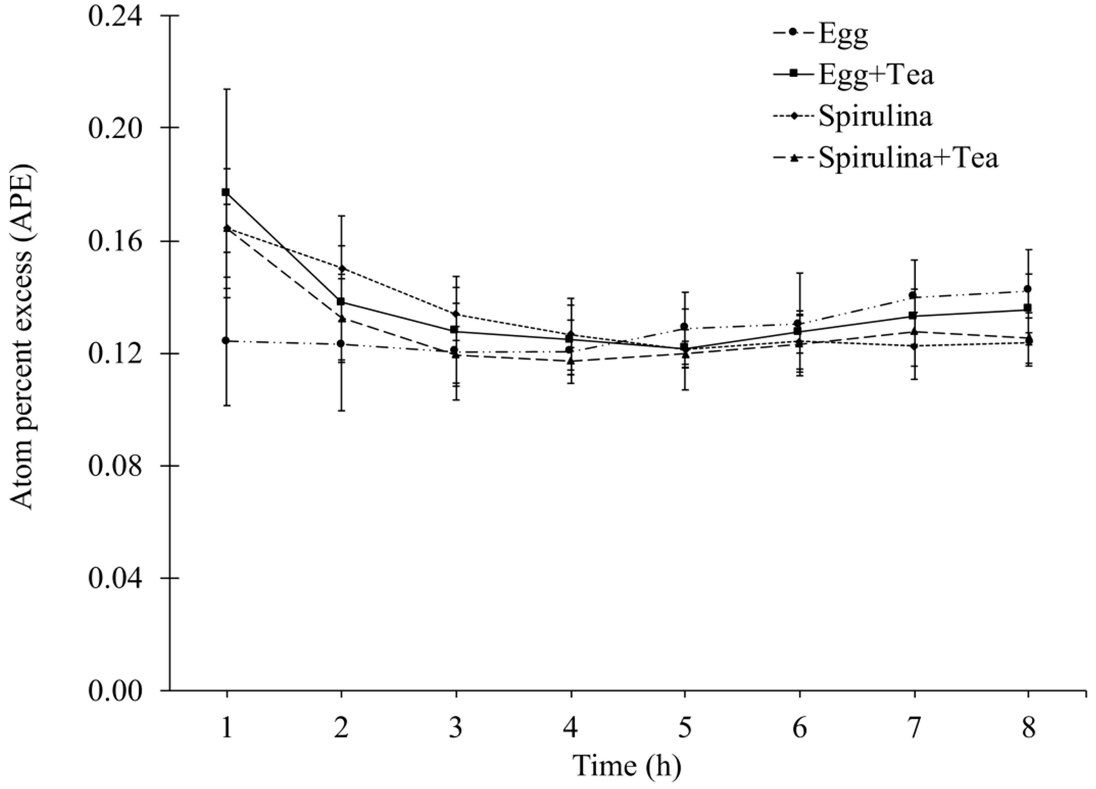


Supplemental Figure 3. Breath $^{13}CO_2$ enrichment after consumption of spirulina with and without tea, and egg with and without tea test meals by healthy Indian adults. Expressed in atom percent excess (APE); represented as mean ± SD (*n*=5 for egg and *n*=3 for spirulina digestibility studies). The breath $^{13}CO_2$ enrichment after consumption of egg without tea test meal is mean ± SD of *n*=5 subjects from a previously published whole boiled egg study*.

* Kashyap S, Shivakumar N, Varkey A, Duraisamy R, Thomas T, Preston T, Devi S, Kurpad AV. Ileal digestibility of intrinsically labeled hen's egg and meat protein determined with the dual stable isotope tracer method in Indian adults. Am J Clin Nutr. 2018 Oct 1;108(5):980-7.
