# Supplementary material for: Co-ingestion of Black Tea Reduces the Indispensable Amino Acid Digestibility of Hens’ Egg in Indian Adults
Source: J Nutr. 2019 May 25;149(8):1363–8. doi: 10.1093/jn/nxz091 (PMC6682489; doi:10.1093/jn/nxz091)
Supplement: nxz091_Supplemental_Files [file nxz091_supplemental_files.zip › Supplemental Table 1.pdf]

Supplementary data

Supplemental Table 1. Composition of U-[^{2}H]-crystalline amino acid mixture[1]

Amino Acids	Concentration %
L-Lysine:2HCl	12
L-Leucine	9
L-Glutamic Acid	9
L-Aspartic Acid	8
L-Alanine	6
L-Arginine:HCl	6
L-Glutamine	5
Glycine	5
L-Asparagine:H_2O	5
L-Proline	5
L-Phenylalanine	4
L-Valine	4
L-Serine	4
L-Threonine	4
L-Tyrosine	3
L-Iso-leucine	3
L-Tryptophan	3
L-Cysteine	3
L-Histidine:HCl:H_2O	1
L-Methionine	1

[1]Adapted from Cambridge Isotope Laboratories, MA, USA (Cell free amino acid mix, 20 AA, U-D 98%, DLM-6819 http://shop.isotope.com/supplyimages/MSDS020/CELL_FREE_AMINO_ACID_MIX__20_AA___U_D__98___DLM_6819_GHS_V.4.0.pdf)
