# Supplementary material for: Co-ingestion of Black Tea Reduces the Indispensable Amino Acid Digestibility of Hens’ Egg in Indian Adults
Source: J Nutr. 2019 May 25;149(8):1363–8. doi: 10.1093/jn/nxz091 (PMC6682489; doi:10.1093/jn/nxz091)
Supplement: nxz091_Supplemental_Files [file nxz091_supplemental_files.zip › Supplemental Table 2.pdf]

Supplementary data

Supplemental Table 2: Meal ^{2}H and ^{13}C IAA enrichments (ppm excess) in test meal of spirulina with and without tea and egg with tea administered to healthy Indian adults.[1]

Amino Acids	Spirulina ± Tea		Egg + Tea	
	^{2}H	^{13}C	^{2}H	^{13}C
	Parts per million excess (ppme)* 10^3			
Methionine	4.71 ± 0.31	0.50 ± 0.02	0.22 ± 0.03	0.43 ± 0.04
Phenylalanine	5.17 ± 0.89	15.11 ± 0.43	2.25 ± 0.31	11.56 ± 1.39
Threonine	4.64 ± 0.07	0.44 ± 0.06	0.48 ± 0.11	0.38 ± 0.01
Lysine	11.29 ± 1.38	19.81 ± 1.82	1.32 ± 0.43	29.33 ± 3.70
Leucine	10.23 ± 3.20	0.56 ± 0.18	0.89 ± 0.31	0.95 ± 0.07
Iso-leucine	6.15 ± 2.28	0.51 ± 0.17	1.51 ± 0.36	1.20 ± 0.12
Valine	8.91 ± 3.42	0.38 ± 0.13	2.30 ± 0.55	0.61 ± 0.07

[1]Values are mean ± SD, *n*=3 for spirulina ± tea experiment and *n*=5 for egg + tea study. The subjects in spirulina ± tea were subset from egg ± tea study
