# Supplementary material for: Co-ingestion of Black Tea Reduces the Indispensable Amino Acid Digestibility of Hens’ Egg in Indian Adults
Source: J Nutr. 2019 May 25;149(8):1363–8. doi: 10.1093/jn/nxz091 (PMC6682489; doi:10.1093/jn/nxz091)
Supplement: nxz091_Supplemental_Files [file nxz091_supplemental_files.zip › Supplemental Table 3.pdf]

Supplementary data

Supplemental Table 3: Plasma ^{2}H and ^{13}C IAA enrichments (ppm excess) at plateau after consumption of spirulina with and without tea and egg with tea in healthy Indian adults.[1]

Amino acids	Spirulina		Spirulina +Tea		Egg + Tea	
	^{2}H	^{13}C	^{2}H	^{13}C	^{2}H	^{13}C
	Parts per million excess (ppme)					
Methionine	289 ± 64	26 ± 4	264 ± 38	26 ± 4	33 ± 7	87 ± 20
Phenylalanine[2]	0.83 ± 0.23	2.23 ± 0.43	1.02 ± 0.27	2.58 ± 0.47	0.34 ± 0.04	1.98 ± 0.27
Threonine	591 ± 139	45 ± 10	534 ± 107	42 ± 6	58 ± 10	52 ± 9
Lysine[2]	1.13 ± 0.27	1.76 ± 0.36	1.63 ± 0.45	2.50 ± 0.66	0.11 ± 0.05	2.39 ± 0.43
Leucine	443 ± 105	21 ± 4	534 ± 112	25 ± 5	111 ± 42	132 ± 13
Isoleucine	375 ± 69	27 ± 6	364 ± 73	25 ± 3	152 ± 56	138 ± 25
Valine	576 ± 86	23 ± 3	703 ± 145	28 ± 8	302 ± 90	98 ± 11

[1]Values are mean ± SD, n=3 for spirulina ± tea study and n=5 for egg + tea study. The subjects in spirulina ± tea were subset from egg ± tea study

[2]Represented as ppme*10^{3}
